# Concomitant Pheochromocytoma and Primary Aldosteronism: A Case Series and Literature Review

**DOI:** 10.1210/jendso/bvab107

**Published:** 2021-06-16

**Authors:** Jimmy J Mao, Jessica E Baker, William E Rainey, William F Young, Irina Bancos

**Affiliations:** 1 Department of Medicine, Mayo Clinic, Rochester, MN, USA; 2 Department of Molecular & Integrative Physiology, University of Michigan, Ann Arbor, MI, USA; 3 Department of Internal Medicine, University of Michigan, Ann Arbor, MI, USA; 4 Division of Endocrinology, Diabetes, Metabolism, and Nutrition, Mayo Clinic, Rochester, MN, USA

**Keywords:** pheochromocytoma, primary aldosteronism, concomitant, adrenal vein sampling, hypertension, hypokalemia

## Abstract

**Context:**

The detection and management of concomitant pheochromocytoma (PHEO) and primary aldosteronism (PA) is not well understood.

**Objective:**

To investigate varying presentations and outcomes of cases with coexisting PHEO and PA to provide an approach to its diagnosis and management.

**Methods:**

We conducted a retrospective case series of adult patients with concomitant PHEO and PA at Mayo Clinic from 2000-2020 and an additional review of cases before 2000 and from the medical literature. Clinical, biochemical, radiologic, and histologic parameters were measured.

**Results:**

Fifteen patients (53% men, median age 53 years) were diagnosed with concomitant PHEO and PA. The majority presented with hypertension (13, 87%) and hypokalemia (13, 87%), and 6 (40%) presented with symptoms suggestive of catecholamine excess. All patients who underwent preoperative workup for catecholamine excess (14, 93%) were found to have biochemical levels above the upper limits of normal. Adrenal vein sampling (AVS) was performed in 9 patients (60%), where 5 (56%) were diagnosed with bilateral PA, and 4 (44%) with unilateral PA. Patients underwent either unilateral (12, 80%) or bilateral (3, 20%) adrenalectomy. Biochemical improvement or resolution of catecholamine excess was confirmed in all cases with documented measurements. Recurrence of PHEO was not observed. Six patients (40%) displayed persistent PA postoperatively.

**Conclusion:**

Concomitant PHEO and PA is a rare but likely underreported condition. Hypertension with or without hypokalemia should prompt evaluation for PA, while any indeterminate adrenal mass should be assessed for PHEO. Coexisting disease warrants consideration of AVS to determine the laterality of PA to ensure appropriate management.

Primary aldosteronism (PA) and catecholamine-secreting pheochromocytomas (PHEOs) are 2 well-known endocrine causes of secondary hypertension. While the prevalence of PHEOs in patients with hypertension is low (0.05%-0.1%) [1], the prevalence of PA is substantially higher, occurring in around 5% of patients with hypertension [2] and 20% of those with treatment-resistant hypertension [3, 4]. The simultaneous occurrence of PHEO and PA is very rare and possibly underreported, with only a handful of case reports published over the last several decades [5-13].

The diagnostic approach to PHEO and PA differs. Although PHEOs may present with symptoms of catecholamine surge such as diaphoresis, palpitations, and headaches in around 30% of cases, they are most frequently discovered incidentally on cross-sectional imaging [14]. In contrast, PA is more commonly discovered during the workup for hypertension. Hypokalemia has been described in up to 28% of patients with PA [15, 16], typically in more severe cases. Both PHEOs and PA can present as unilateral or bilateral adrenal disease, and diagnosing them simultaneously may be difficult. The management of coexisting PHEO and PA is also challenging. If a PHEO is not properly diagnosed initially, an adrenalectomy for a presumed aldosterone-producing adenoma without preoperative adrenergic blockade [11] can result in severe hypertensive crisis [17, 18], which may lead to life-threatening cerebrovascular hemorrhage or myocardial infarction. Previous management approaches have included a unilateral adrenalectomy for a suspected PHEO, with subsequent medical therapy for the coexisting PA when the cure for PA was not achieved by the initial adrenalectomy, such as in cases with contralateral or bilateral aldosterone excess [10, 12].

Much remains unknown of this rare condition, and a more refined understanding of the case detection, diagnosis, and management of coexisting PHEO and PA is needed. Our objectives were to describe the clinical, biochemical, radiologic, and histologic characteristics of cases with confirmed concomitant PHEO and PA and to follow posttreatment outcomes in order to provide an approach to diagnosis and management.

## Materials and Methods

This was a retrospective study of adult patients (age ≥18 years) with concomitant PHEO and PA evaluated at the Mayo Clinic in Rochester, Minnesota. This study was approved by the Mayo Clinic Institutional Review Board. A literature search for additional case reports describing coexisting PHEO and PA was also performed.

### Patients

We identified eligible patients through 3 approaches. First, we utilized an existent longitudinal adrenal mass database of 7724 patients from 2000-2020 at Mayo Clinic. A computerized search function was used to identify study participants with previous documentation of PHEO and case-detection testing for PA. Subsequent medical record review was performed to confirm the diagnosis of both PHEO and PA for each case of interest. The diagnosis of PHEO was based on histopathology, and the diagnosis of PA was based on biochemical case-detection guidelines [19]. Second, 2 patients known to and treated by a co-investigator and diagnosed with coexisting disease prior to 2000 were also included in our study population. In addition, a literature search for additional case reports describing concomitant PHEO and PA was performed.

Clinical variables were collected from medical records (for patients identified as part of the retrospective study) and from the published case reports. Variables of interest included: sex; age at diagnosis; presence of symptoms suggestive of catecholamine excess at presentation (ie, headaches, palpitations, diaphoresis, and/or anxiety spells); blood pressure and serum potassium levels at presentation; biochemical measurements of plasma and urinary fractionated catecholamines and/or metanephrines (and urinary vanillylmandelic acid); biochemical phenotypes of PHEOs; screening testing for PA with plasma aldosterone concentration (PAC) and plasma renin activity (PRA); confirmatory testing for PA with 24-hour urinary aldosterone excretion; tumor size(s) based on imaging; site of aldosterone excess on adrenal vein sampling (AVS) if performed; type of adrenalectomy and associated histopathology; postoperative biochemical outcomes of fractionated catecholamines and/or metanephrines, urinary vanillylmandelic acid, PAC, and PRA; and postoperative blood pressure and serum potassium levels. Hypertension was defined as having a systolic blood pressure (SBP) ≥140 mm Hg, diastolic blood pressure (DBP) ≥90 mm Hg, and/or requiring antihypertensive therapy. Hypokalemia was defined as having a serum potassium concentration <3.5 mEq/L and/or requiring potassium supplementation or mineralocorticoid receptor blockade. PAC and PRA were measured via liquid chromatography-tandem mass spectrometry at Mayo Clinic. A positive case-detection test for PA was defined by a PAC ≥10 ng/dL and a PRA ≤1 ng/mL/h. A positive confirmatory test for PA was defined by a 24-hour urinary aldosterone excretion >12 mcg after oral sodium loading [19]. Positivity for the above tests was also defined by the clinical documentation of a medical provider describing biochemical results as consistent with PA when the numerical values were not recorded. The biochemical phenotypes of PHEOs were classified as adrenergic (epinephrine and metanephrine predominance), noradrenergic (norepinephrine and normetanephrine predominance), and/or dopaminergic (dopamine and methoxytyramine predominance).

### Immunohistochemistry

Tissue samples of 8 adrenal glands from the 5 included patients evaluated and treated at our institution were analyzed with immunohistochemistry. Formalin-fixed, paraffin-embedded tissue sections were cut to a thickness of 5 µm. Immunohistochemistry was performed using primary antibodies against aldosterone synthase (CYP11B2) and tyrosine hydroxylase as markers for aldosterone and catecholamine producing cells, respectively. The antibodies used in immunohistochemistry were as follows: mouse monoclonal antibody against CYP11B2 (MABS1251 from Millipore/Sigma, 1:1250) [20] and a rabbit polyclonal antibody against tyrosine hydroxylase (Provided by Dr. John Porter, 1:15 000) [21]. The reacted slides were counterstained with Harris hematoxylin and then dehydrated and coverslipped.

### Statistics

Categorical data were summarized as numbers and percentages, while continuous data were summarized as medians and ranges.

## Results

### Patients

**Figure 1.**
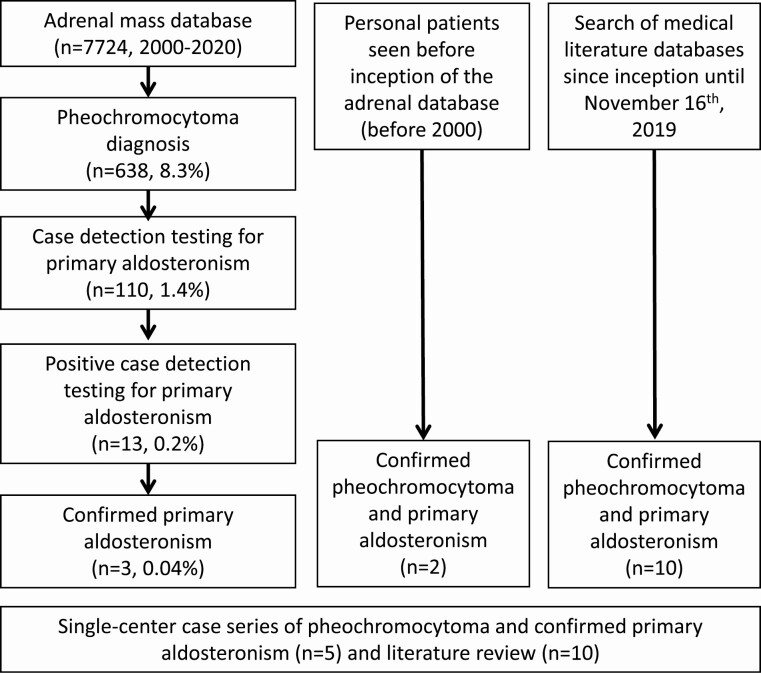
Flowchart for patient selection.

Out of 7724 patients with adrenal tumors, 638 (8.3%) were documented as having PHEO, of whom 110 (17.2%) also underwent biochemical screening for PA with measurements of PRA and PAC. Of the 110 patients with PHEO who were screened for PA, 13 (11.4%) had a positive case-detection test, of whom 3 were confirmed to have PA. Of the 110 patients with PHEO, 102 underwent PA screening for the following reasons: workup for adrenal incidentaloma (76%), workup for suspected secondary causes of hypertension (22%), and other (2%). Two patients who were seen prior to 2000 by one co-investigator met inclusion criteria and were also included, culminating in a total of 5 patients (patients 1-5) with concomitant PHEO and PA from our single-center retrospective study, Fig. 1. Ten additional case reports were included from our literature search [5-13], which correspond to patients 6 to 15. No patients were documented to have an underlying genetic syndrome.

### Single-Center Case Series

#### Clinical, biochemical, and imaging presentation

Of the 5 patients with confirmed PHEO and PA (4 men, 1 woman), median age at diagnosis was 62 years (range, 59-68). Initial diagnostic evaluation for adrenal pathology was prompted by the presence of hypertension, symptoms suggestive of catecholamine excess, and/or the presence of hypokalemia (Table 1). All 5 initially presented with hypertension, with 4 (80%) also presenting with symptoms suggestive of catecholamine excess. All were taking antihypertensive medications (range, 1-5 medications) with 4 demonstrating suboptimal blood pressure control (SBP ≥140 mm Hg and/or DBP ≥90 mm Hg). All patients presented with hypokalemia; 2 were treated with potassium supplementation and 2 were treated with mineralocorticoid receptor antagonists. The median PAC was 22 ng/dL (range, 11-45; normal < 10), and the median PRA was 0.6 ng/mL/h (range, 0.6-0.7; normal > 1), as detailed in Table 1. All patients also displayed biochemical evidence of catecholamine excess, with plasma and/or 24-hour urinary fractionated catecholamines and/or metanephrines above the upper limits of normal.

**Table 1.** Diagnostic characteristics of Mayo Clinic and case-reported patients with coexisting pheochromocytoma and primary aldosteronism

Patient number	Sex	Age (years)	Symptoms of catecholamine excess^a^	Initial blood pressure (mm Hg)	Hypokalemia^b^ present	PAC^c^ (ng/dL)	PRA^d^ (ng/mL/h)	Urinary aldosterone^e^ (mcg/24 h)	Biochemical phenotype of PHEO	Tumor size(s) on imaging (mm)	Site of aldosterone excess on adrenal vein sampling
1	Male	59	Yes	142/86	Yes	45	0.6	35	Adrenergic, Dopaminergic	Right: 30	Bilateral
2	Male	68	No	170/90	Yes	29	0.6	31	Adrenergic	Right: 33	Not performed
3 [22]	Male	63	Yes	160/94	Yes	22	0.7	29.2	Adrenergic	Right: 40	Bilateral
										Left: 12	
4	Male	62	Yes	160/75	Yes	18	0.6	40	Noradrenergic	Right: 10	Bilateral
										Left: 12	
5	Female	62	Yes	124/84	Yes	11	0.6	84	Noradrenergic	Right: 31	Not performed
										Left: 44	
6 [5]	Male	49	No	150/110	Yes	28.5	0.21	Unknown	Functioning, phenotype unclear	No imaging	Left
7 [6]	Male	46	No	180/100	Yes	20	0.13	Unknown	Noradrenergic	Right: unknown	Not performed
8 [7]	Female	40	No	“Mildly hypertensive”	Yes	21.4	0.3	Unknown	Adrenergic	Right: 40	Bilateral (left dominant)
9 [7]	Female	63	Yes	“Normo-tensive”	No	Unknown	Unknown	Unknown	Adrenergic	Left: 50	Not performed
10 [8]	Female	39	No	142/95	Yes	64	0.2	Unknown	Unknown	Right: 15	Not performed
11 [9]	Male	49	No	162/96	Yes	117.1	0.1	39.2	Noradrenergic	Right: 40	Left
										Left: 10	
12 [10]	Male	57	No	210/120	Yes	42	0.004	Unknown	Adrenergic	Right: 70	Left
										Left: 10	
13 [11]	Female	40	No	152/92	Yes	20	0.1	Unknown	Noradrenergic, dopaminergic	Left: 25	Left
14 [12]	Female	36	No	202/120	Yes	40.7	1.2	19	Noradrenergic	Right: unknown	Bilateral
										Left: unknown	
15 [13]	Female	53	Yes	150/100	No	33.4	0.005	Unknown	Adrenergic	Right: 30	Not performed
										Left: 20, 7	

Abbreviations: PAC, plasma aldosterone concentration; PHEO, pheochromocytoma; PRA, plasma renin activity.

^a^Symptoms of catecholamine excess were defined as headaches, palpitations, diaphoresis, and/or anxiety spells.

^b^Hypokalemia was defined as K <3.5 mEq/L and/or requiring potassium supplementation or mineralocorticoid receptor blockade.

^c^PAC normal range <10 ng/dL;

^d^PRA normal range <1 ng/mL/h;

^e^Normal range of urinary aldosterone concentration was <12 mcg/24 h.

**Figure 2.**
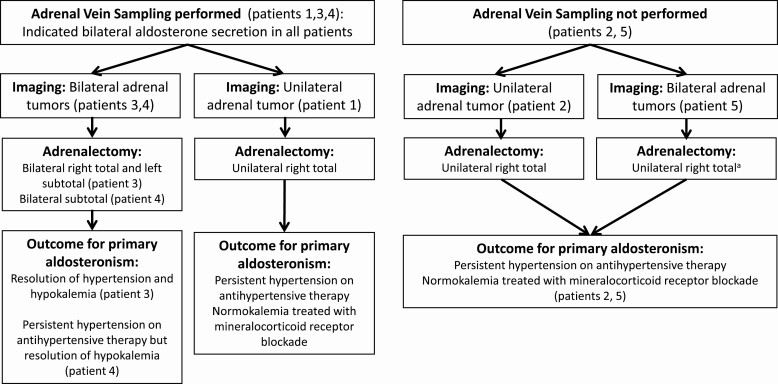
Diagnostic workup, management, and outcomes in the single-center series of 5 patients. ^a^Patient 5 required lifelong corticosteroid and mineralocorticoid replacement after undergoing left total adrenalectomy for subclinical glucocorticoid secretory autonomy 3 years after initial right total adrenalectomy.

Abdominal imaging revealed unilateral adrenal tumors in 2 patients (patients 1 and 2) and bilateral adrenal tumors in 3 patients (patients 3, 4, and 5). The median maximum tumor diameter was 33 mm (range, 12-44). AVS was performed in 3 patients (patients 1, 3, and 4), and demonstrated bilateral aldosterone excess in all 3 cases (Fig. 2, Table 1).

#### Histopathology and therapeutic interventions

Adrenalectomies were performed in 4 patients to treat PHEO and in 1 patient to treat PA (Fig. 2, Table 2). Histologic/pathologic examination using hematoxylin and eosin, tyrosine hydroxylase, and CYP11B2 immunohistochemistry was performed with tissue sections from 16 formalin-fixed, paraffin-embedded blocks prepared from 8 adrenals from the 5 PHEO and PA cases (patients 3, 4, and 5 with bilateral samples and patients 1 and 2 with right-sided samples). All adrenals had tyrosine hydroxylase–positive immunoreactivity within PHEO and, when present, normal adrenal medulla but not within adrenocortical cells.

**Table 2.** Therapeutic interventions and outcomes of Mayo Clinic and case-reported patients with coexisting pheochromocytoma and primary aldosteronism

Patient number	Type of adrenalectomy	Final histopathology	Postoperative catecholamine excess improved	Postoperative biochemical outcome of PA^a^	Postoperative hypertension^b^	Postoperative potassium (mEq/L)
1	Right total	**Right:** 3.8 cm composite PHEO with focal areas of ganglioneuroblastoma differentiation and hemorrhage, a discrete cortical nodule in the zona glomerulosa	Yes	PAC: 16	**Yes**	Potassium normal
				PRA: 0.6	BP normal	On MRA
				Bilateral PA on AVS	On anti-hypertensives	
				**Persistent PA**		
2	Right total	**Right:** 3.5 cm PHEO	Yes	PAC: 14	**Yes**	Potassium 4.2
				PRA: 0.6	BP 170/80	On MRA
				**Persistent PA**	On anti-hypertensives	
3 [22]	Right total and left subtotal	**Right:** 2 PHEOs (4 cm and 0.9 cm); mild cortical hyperplasia with focal occurrence of cells with deeply eosinophilic cytoplasm in the superficial cortex, nuclear enlargement, and cytoplasmic globules, reminiscent of aldactone bodies; 3 mm cavernous hemangioma also present in cortex	Yes	PAC: 18	**No**	Potassium normal
				PRA: 1.2	BP 122/80	Not on MRA
				24-hour urinary aldosterone: 2.7 mcg	Not on anti-hypertensives	
				**Resolved PA**		
		**Left:** 1.5 cm PHEO; mild cortical hyperplasia with 2 cortical nodules				
4	Bilateral subtotal	**Right:** 1.5 cm PHEO; multinodular cortical hyperplasia, with multiple nodules up to 0.8 cm	Yes	PAC: 2	**Yes**	Potassium 4.2
				PRA: normal	BP 120/70	Not on MRA
		**Left:** Multinodular cortical hyperplasia, with nodules up to 1.0 cm				
				**Resolved PA**	On anti-hypertensives	
5	Right total	**Right:** 2.5 cm PHEO, R macronodular hyperplasia of adrenal cortex	Yes	PAC: 13	**Yes**	Potassium 5.1
				PRA: 0.2	BP 180/100	On MRA
				**Persistent PA**	On anti-hypertensives	
		Left total adrenalectomy^c^ was performed years later due to autonomous cortisol secretion, which showed diffuse and nodular adrenocortical hyperplasia				
6 [5]	Left total	**Left:** 0.7 cm PHEO; 2 cm cortical tumor; 0.2 cm and 0.1 cm cortical nodule composed of lipid laden clear cells. No hyperplastic changes in remaining cortex, only focal lymphocytic and plasma cell infiltration at the zona glomerulosa	Yes	PAC: 10.4	**Unknown**	Potassium normal
				PRA: 3.2	BP 130/80	Unknown if on MRA
				**Resolved PA**	Unknown if on anti-hypertensives	
7 [6]	Right total	**Right:** Adrenal medulla totally occupied by PHEO; 1.0 cm adrenocortical adenoma	Yes	PAC: 5	**Unknown**	Unknown potassium or if on MRA
				PRA: unknown	BP normal	
				**Resolved PA**	Unknown if on anti-hypertensives	
8 [7]	Right total	**Right:** 3.3 cm PHEO extending up to the capsule and merging with a nodular hyperplastic area of zona fasciculata; in other areas of cortex, hyperplasia of zona glomerulosa was seen; karyotype abnormalities of long-term tissue culture of cortical cells	Yes	PAC: unknown	**Unknown**	Unknown potassium or if on MRA
				PRA: unknown	BP normal	
				Fludrocortisone failed to suppress PAC	Unknown if on anti-hypertensives	
				Bilateral PA on AVS		
				**Persistent PA**		
9 [7]	Left total	**Left:** 5.5 cm PHEO; 1 cm cortical adenoma with many other areas of hyperplastic adrenal cortex including nodules up to 0.3 cm diameter which histologically resembled zona fasciculata	Yes	PAC: unknown	**Unknown**	Unknown potassium or if on MRA
				PRA: unknown	Unknown BP or if on anti-hypertensives	
				Normal PAC/PRA		
				**Resolved PA**		
10 [8]	Right total	**Right:** mass involved both cortex and medulla and was consistent with PHEO and adrenal cortical adenoma	Unknown	Blood pressure and hypokalemia resolved	**No**	Potassium 4.2
				**Resolved PA**	BP 110/60	Not on MRA
					Not on anti-hypertensives	
11 [9]	Bilateral subtotal	**Right:** 5.0 cm PHEO; nonnodular yellow cortical layer contained an adrenocortical adenoma with clear cells in a honeycomb pattern	Yes	PRA: normal	**Unknown**	Unknown potassium or if on MRA
				PAC: normal	BP normal	
				**Resolved PA**	Unknown if on anti-hypertensives	
		**Left:** 1.9 cm yellowish mass that was consistent with adrenocortical adenoma composed of clear cells				
12 [10]	Right total	**Right:** 10 cm PHEO	Unknown	Previous AVS suggested left-sided PA	**Yes**	Potassium normal
					BP normal	
				Treated with right adrenalectomy for PHEO	On anti-hypertensives	On MRA
				**Persistent PA**		
13 [11]	Left total	**Left:** 1.9 cm PHEO; 2.4 cm yellow round mass composed of clear cells, P450C17 not expressed (suggesting tumor not secreting cortisol) though all other steroid synthetases (p450SCC, 3BHSD, p450c21, and p450c11) were expressed, suggesting aldosterone production. Zona glomerulosa of normal adrenal gland showed hyperplasia with no expression of 3B-HSD, which was construed as a finding of paradoxical hyperplasia associated with aldosterone overproduction. No significant atrophy in zona fasciculata or reticularis (no HPA axis suppression so likely no long-term cortisol production)	Yes	PAC: 4.28	**No**	Potassium 4
				PRA: 0.3	BP 120/70	Not on MRA
				**Resolved PA**	Not on anti-hypertensives	
14 [12]	Right total	**Left:** 2.2 cm PHEO, 1.1 cm yellowish tumor composed of lipid laden clear cells, diagnosed as adrenocortical adenoma (mRNA evidence of aldosterone production	Yes	PAC: 52.7	**Yes**	Potassium 3
				PRA: 0.1	BP 120/70	On MRA
				Bilateral PA on AVS	On anti-hypertensives	
				**Persistent PA**		
15 [13]	Right total	**Right:** 4 cm PHEO	Yes	PAC: unknown	**No**	Unknown potassium
				PRA: unknown	BP normal	Not on MRA
				Normal PAC/PRA	Not on anti-hypertensives	
				**Resolved PA**		

Abbreviations: MRA, mineralocorticoid receptor antagonist; PA, primary aldosteronism; PAC, plasma aldosterone concentration; PHEO, pheochromocytoma; PRA, plasma renin activity.

^a^Postoperative biochemical outcomes of PA described persistence or resolution of PA; PAC normal range <10 ng/dL; PRA normal range <1 ng/mL/h.

^b^Hypertension was defined as systolic blood pressure (SBP) ≥140 mm Hg, diastolic blood pressure (DBP) ≥90 mm Hg, and/or requiring antihypertensive therapy.

^c^Patient 5 required lifelong corticosteroid and mineralocorticoid replacement after undergoing left total adrenalectomy for subclinical glucocorticoid secretory autonomy 3 years after initial right total adrenalectomy.

**Figure 3.**
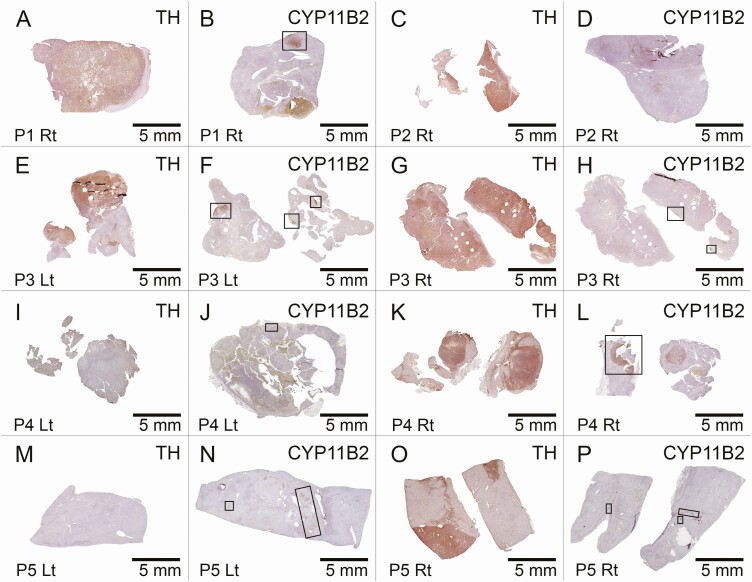
Immunohistochemistry findings of the 5 patients in the single-center series. Tyrosine hydroxylase (TH) and aldosterone synthase (CYP11B2) immunohistochemistry findings of the adrenal glands from the 5 patients with coexisting PHEO and PA are shown. Adrenal sections from patients 1-5 (P1-P5) were used for TH (Panels A, C, E, G, I, K, M and O) and CYP11B2 (Panels B, D, F, H, J, L, N, P) immunohistochemistry. P1 and P2 underwent unilateral adrenalectomy. P3, P4, and P5 underwent bilateral adrenalectomy. Representative images from multiple formalin-fixed, paraffin-embedded sections are shown for the right (Rt) and left (Lt) adrenals. Scale bars, 5 mm. Boxes represent aldosterone-producing nodules or aldosterone-producing cell clusters.

Patient 1 was found to have a unilateral adrenal mass concerning for a PHEO and treated with a right total adrenalectomy. Pathology confirmed a tyrosine hydroxylase–positive PHEO with marked hemorrhage, and an adjacent discrete CYP11B2-positive cortical nodule in the zona glomerulosa (Fig. 3, Panels A and B). Biochemical resolution of catecholamine excess was confirmed postoperatively. He demonstrated persistent PA (PRA of 0.6 ng/mL/h and PAC of 16 ng/dL) following unilateral adrenalectomy. He required antihypertensive therapy to control his blood pressure. Serum potassium levels were normal, but he required mineralocorticoid receptor blockade following surgery.

Patient 2 was also found to have a unilateral adrenal mass concerning for a PHEO and treated with a right total adrenalectomy. Pathology confirmed a tyrosine hydroxylase–positive PHEO but without concomitant CYP11B2-postive cortical nodules or hyperplasia (Fig. 3, Panels C and D), possibly indicating a contralateral aldosterone-secreting lesion. Postoperatively, although biochemical resolution of catecholamine excess was confirmed, the patient continued to demonstrate biochemical evidence of persistent PA (PRA of 0.6 ng/mL/h and PAC of 14 ng/dL). He remained hypertensive (SBP ≥170 mm Hg), requiring multiple antihypertensive medications, including mineralocorticoid receptor blockade to control potassium levels following surgery.

Patient 3, who was previously reported in the literature [22], was found to have bilateral adrenal tumors concerning for bilateral PHEOs and underwent a right total and left subtotal adrenalectomy. Pathology confirmed bilateral tyrosine hydroxylase–positive PHEOs (Fig. 3, Panels E and G). The left adrenal exhibited a discrete CYP11B2-positive nodule, and both adrenals exhibited aldosterone-producing cell clusters (Fig. 3, Panels F and H). Postoperatively, fractionated catecholamines and/or metanephrines, PRA (1.2 ng/mL/h), PAC (1.8 ng/dL), and 24-hour urinary aldosterone (2.7 mcg) normalized, consistent with resolved PA. Blood pressure and serum potassium levels also normalized without pharmacotherapy. There was no biochemical evidence of recurrent PA for at least 18 years following surgery.

Patient 4 was found to have bilateral adrenal tumors thought to represent aldosterone-producing adenomas without initial suspicion for PHEO. Preoperative catecholamines showed a normal metanephrine level at 0.34 nmol/L (normal <0.5 nmol/L) and mildly elevated normetanephrine level at 1.36 nmol/L (normal <0.9 nmol/L). Due to spironolactone intolerance, the patient underwent bilateral subtotal adrenalectomy. Pathology and immunohistochemistry revealed a right adrenal tyrosine hydroxylase–positive PHEO and CYP11B2-positive adenoma (Fig. 3, Panels K and L). The left adrenal had cortical hyperplasia with a CYP11B2-positive nodule but no tyrosine hydroxylase–positive cells (Fig. 3, Panels I and J). Intraoperative and postoperative courses were uncomplicated. Following surgery, catecholamines and/or metanephrines, PRA, and PAC (2 ng/dL) normalized, consistent with resolved PA. Blood pressure and serum potassium levels remained normal with antihypertensive therapy without mineralocorticoid receptor blockade. Nearly 14 years after surgery, PAC was 6.2 ng/dL and PRA was 2.3 ng/mL/h, demonstrating no biochemical recurrence of PA.

Patient 5 was also found to have bilateral adrenal tumors. As the right adrenal mass was concerning for PHEO, she was initially treated with a right total adrenalectomy. Pathology confirmed a right tyrosine hydroxylase–positive PHEO and nodular cortical hyperplasia with multiple CYP11B2-positive aldosterone-producing cell clusters (Fig. 3, Panels O and P). Postoperatively, fractionated catecholamines and/or metanephrines normalized, but the patient displayed biochemical evidence of persistent PA (PRA of 0.2 ng/mL/h and PAC of 13 ng/dL). Potassium levels remained normal on mineralocorticoid receptor blockade, but she remained hypertensive (SBP ≥180 mm Hg) despite being on multiple antihypertensive medications. She subsequently underwent a left total adrenalectomy 3 years later due to persistent subclinical glucocorticoid secretory autonomy, where pathology revealed diffuse nodular cortical hyperplasia that included diffuse CYP11B2-positive cells (Fig. 3, Panels M and N). She required lifelong glucocorticoid and mineralocorticoid replacement thereafter.

### Combined Single-Center Case Series and Literature Review

Of all 15 patients with concomitant PHEO and PA (8 men, 53%), median age at diagnosis was 53 years (range, 36-68). Initial diagnostic evaluation for adrenal pathology was prompted by the presence of hypertension, symptoms suggestive of catecholamine excess, and/or the presence of hypokalemia (Table 1). Patients 9 and 15 also presented with abdominal pain. Patients 11 and 14 were known to have adrenal masses at presentation, but the reasons for how these were diagnosed were not described. The vast majority presented with hypertension (13, 87%) and hypokalemia (13, 87%), and 6 (40%) presented with symptoms suggestive of catecholamine excess. Of the 13 patients who presented with hypertension, 12 presented with hypokalemia. One patient was found to be “mildly hypertensive” on presentation, and another patient presented with normotension. The median PAC was 28.8 ng/dL (range, 11-117.1), and the median PRA was 0.255 ng/mL/h (range, 0.004-1.2), as shown in Table 1. All patients with preoperative workup for catecholamine excess (14, 93%) were found to have elevated plasma and/or urinary fractionated catecholamines and/or metanephrines (or elevated urinary vanillylmandelic acid) above the upper limits of normal. Of these 14 patients, 6 (43%) were found to have an adrenergic biochemical phenotype, 5 (36%) were found to have a noradrenergic biochemical phenotype, 1 (7%) was found to have a mixed adrenergic and dopaminergic biochemical phenotype, 1 (7%) was found to have a mixed noradrenergic and dopaminergic biochemical phenotype, and 1 (7%) was found to have an unclear functioning phenotype (elevated urinary vanillylmandelic acid only).

Of the 14 patients who underwent imaging, 7 (50%) were found to have unilateral adrenal tumors, and 7 (50%) were found to have bilateral adrenal tumors. Median maximum tumor diameter was 36.5 mm (range, 12-70). Of the 9 patients who underwent AVS, 5 (56%) were diagnosed with bilateral PA, and 4 (44%) were diagnosed with left-sided PA, of which 2 were ipsilateral to the PHEO and 2 were contralateral to the PHEO (Table 1). All patients underwent either unilateral (12, 80%) or bilateral (3, 20%) adrenalectomy to treat their PHEO and/or PA. Of the 14 patients initially found to have biochemical evidence of catecholamine excess, postoperative fractionated catecholamines and/or metanephrines (or urinary vanillylmandelic acid) normalized or improved in 13 (93%) and was not measured in 1 patient. Recurrence of PHEO was not observed in any cases.

A total of 6 patients (40%; patients 1, 2, 5, 8, 12, and 14) displayed persistent PA postoperatively (Table 2). Three (patients 1, 8, and 14) were found to have bilateral PA on AVS but underwent unilateral right-sided total adrenalectomy to treat their PHEO, logically resulting in persistent PA following surgery. One (patient 12) was found to have left-sided PA on AVS but underwent right-sided total adrenalectomy to treat his PHEO, logically resulting in persistent PA following surgery. Two (patients 2 and 5) did not undergo AVS but were found to have persistent PA following unilateral right-sided total adrenalectomy to treat PHEO, suggesting prior left-sided or bilateral aldosterone excess. Of the 6 patients with persistent postoperative PA, 5 required long-term mineralocorticoid receptor blockade, and 1 did not have documentation of requiring mineralocorticoid receptor blockade (Table 2).

## Discussion

In our study of 15 patients with coexisting PHEO and PA, 80% initially presented with both hypertension and hypokalemia, but only 40% displayed symptoms suggestive of catecholamine excess. All patients with preoperative catecholamine-related workup demonstrated biochemical excess with plasma and/or urinary fractionated catecholamines and/or metanephrines (or urinary vanillylmandelic acid) above the upper limits of normal; postoperatively, all measured fractionated catecholamines and/or metanephrines (or urinary vanillylmandelic acid) normalized or improved, and no recurrence of PHEO was observed. AVS was performed in 60% of patients (bilateral aldosterone excess in 56%, ipsilateral aldosterone excess to PHEO in 22%, contralateral aldosterone excess to PHEO in 22%). Six patients were found to have persistent PA following unilateral adrenalectomy to treat PHEO, where 5 required long-term mineralocorticoid receptor blockade, and 1 did not have documentation of requiring mineralocorticoid receptor blockade.

### Presentation and Diagnosis

We found that 80% of our patients with concomitant PHEO and PA initially presented with both hypertension and hypokalemia. This observed presentation of a more severe phenotype of PA may suggest selection bias, as known rates of hypokalemia in PA are significantly lower [15, 16]. It is thus possible that the prevalence of coexisting PHEO and PA is higher than reported because the workup for PA is typically not pursued in a patient with an adrenal mass suspected and confirmed to be a PHEO. While classically known as a disease to present with adrenergic symptoms such as headaches, palpitations, diaphoresis, and/or anxiety spells, PHEOs have more recently been found to be incidentally discovered on imaging in the majority of cases, with only around 30% of diagnoses based on preceding symptoms of catecholamine excess [14]. Our findings appear to support this, as we observed only a minority (40%) of patients with coexisting PHEO and PA initially presenting with symptoms suggestive of catecholamine excess. Therefore, we suggest that all patients presenting with unexplained hypertension with or without hypokalemia should be screened for PA, while PHEO must be considered in any patient found to have an indeterminate adrenal mass (eg, unenhanced computed tomography attenuation >10 Hounsfield units).

### Management Implications

Our patients were treated with either unilateral (80%) or bilateral (20%) adrenalectomy to target their PHEO and/or PA. While undergoing left adrenalectomy for presumed PA, patient 13 [11] was noted to have a dramatic increase in blood pressure to 200/120 mm Hg intraoperatively, suggestive of a concomitant PHEO that was not previously suspected. Fortunately, the adrenal gland was safely removed, with subsequent pathology confirming coexisting disease and resulting in the cure of both PHEO and PA. Due to the observed significant risk of intraoperative hypertensive crisis [17, 18] in the absence of preoperative adrenergic blockade, the safest management approach for patients with suspected coexisting PHEO and PA involves strategic preparation and initial removal for the suspected PHEO, followed by medical therapy for the concurrent PA if the cure for PA was not achieved by the initial adrenalectomy, as seen in patients 12 and 14 with contralateral and bilateral aldosterone excess, respectively [10, 12].

Of the 6 patients (40%) who displayed persistent PA postoperatively, 3 were found to have bilateral PA on AVS but underwent unilateral right-sided adrenalectomy to treat their PHEO, logically resulting in persistent PA following surgery; 1 was found to have left-sided PA on AVS but underwent right-sided adrenalectomy to treat his PHEO, logically resulting in persistent PA following surgery; and 2 did not undergo AVS but were found to have persistent PA following unilateral right-sided adrenalectomy to treat their PHEO, suggesting prior left-sided or bilateral aldosterone excess. We also found a number of cases (patients 7, 10, and 15) [6, 8, 13], where PA resolved following an isolated right adrenalectomy to treat their PHEO and/or PA. Additionally, of the 9 patients who underwent AVS, 5 (56%) were diagnosed with bilateral PA and 4 (44%) with left-sided PA, Table 1. Despite our small sample size, these findings suggest that PA in the setting of concomitant PHEO may present unilaterally or bilaterally without a skewed tendency for one or the other, and regardless of the laterality of the PHEO. Therefore, to ensure appropriate management, AVS may be needed to clearly delineate the laterality of PA in patients with concurrent disease.

### Pheochromocytoma and Primary Aldosteronism

In the medical literature, PHEOs have been reported to ectopically produce various hormones (ie, adrenocorticotropic hormone, corticotropin releasing hormone, parathyroid hormone, vasoactive intestinal polypeptide, growth hormone releasing hormone) resulting in secondary hormone excess syndromes. We observed 2 cases (patients 3 and 4) that demonstrated resolution of AVS-confirmed bilateral PA following bilateral subtotal adrenalectomy (allowing for adequate postoperative production of aldosterone and cortisol), suggesting the possibility of an aldosterone stimulating factor that might have been secreted by the PHEO. However, we did not observe any cases in which PA resolved following unilateral adrenalectomy for PHEO, where AVS had initially demonstrated bilateral aldosterone excess (patients 1, 8, and 14). Therefore, it remains unclear if there is any noncoincidental relationship between these 2 conditions that could be causing excessive aldosterone production.

### Strengths/Limitations

This was the largest case series to date in characterizing the presentations and outcomes of patients with concomitant PHEO and PA. Enrollment of a consecutive patient cohort at our institution allowed for data sampling to occur thoroughly with a degree of standardization in diagnostic evaluation and disease management. As part of a retrospective cohort study, our conclusions were limited to observed associations and susceptible to selection bias and confounding. Given the rare nature of this condition, our sample size was also notably small at 15, making it difficult to find a definitive physiologic or anatomic relationship between PHEO and PA. Additionally, data for certain biochemical variables of interest were missing due to a lack of documentation in earlier medical records. Finally, as our study population spanned across multiple decades, we found considerable heterogeneity in the diagnostic workup and management of this disease, as well as a lack in documentation of long-term outcomes.

## Conclusion

Concomitant PHEO and PA is a rare but likely underreported condition with only a handful of case reports published over the last several decades. A previous history of unexplained hypertension with or without hypokalemia should prompt evaluation for PA. However, in any patient with an indeterminate adrenal mass, PHEO needs to be considered. The appropriate diagnosis and management of coexisting disease warrant consideration of AVS to determine the laterality of PA, preoperative alpha-adrenergic blockade for initial removal of PHEO, and possibly subsequent subtotal adrenalectomy or lifelong mineralocorticoid receptor blockade for those with bilateral or contralateral aldosterone excess.

## Data Availability

Some or all datasets generated during and/or analyzed during the current study are not publicly available but are available from the corresponding author on reasonable request.

## Abbreviations

AVS: adrenal vein sampling
DBP: diastolic blood pressure
PA: primary aldosteronism
PAC: plasma aldosterone concentration
PHEO: pheochromocytoma
PRA: plasma renin activity
SBP: systolic blood pressure

## Additional Informations

***Disclosures:*** Irina Bancos reports consulting with HRA Pharma, Sparrow Pharmaceutics, and Strongbridge outside the submitted work.
